# Ultrafast intramolecular proton transfer reactions and solvation dynamics of DMSO

**DOI:** 10.1063/1.5129446

**Published:** 2019-12-12

**Authors:** Myungsam Jen, Kooknam Jeon, Sebok Lee, Sunjoo Hwang, Won-jin Chung, Yoonsoo Pang

**Affiliations:** Department of Chemistry, Gwangju Institute of Science and Technology, 123 Cheomdangwagi-ro, Buk-gu, Gwangju 61005, South Korea

## Abstract

Ultrafast intramolecular proton transfers of 1,2-dihydroxyanthraquinone (alizarin-h_2_) and its deuterated product (alizarin-d_2_) in dimethyl sulfoxide (DMSO) have been investigated by femtosecond stimulated Raman spectroscopy. The population dynamics in the solute vibrational mode of ν_C=O_ and the coherent oscillations observed in all of the skeletal vibrational modes ν_C=O_ and ν_C=C_ clearly showed the ultrafast excited-state intramolecular proton transfer dynamics of 110 and 170 fs for alizarin-h_2_ and alizarin-d_2_, respectively. Interestingly, we have observed that the solvent vibrational modes ν_S=O_ and ν_CSC_ may also represent ultrafast structural dynamics at the frequencies for its “free” or “aggregated” species. From the kinetic analysis of the ν_S=O_ and ν_CSC_ modes of DMSO, the ultrafast changes in the solvation or intermolecular interactions between DMSO molecules initiated by the structural changes of solute molecules have been thoroughly investigated. We propose that the solvent vibrational modes ν_S=O_ and ν_CSC_ of DMSO can be used as a “sensor” for ultrafast chemical reactions accompanying the structural changes and subsequent solute-solvent interactions.

## INTRODUCTION

**FIG. 1.**
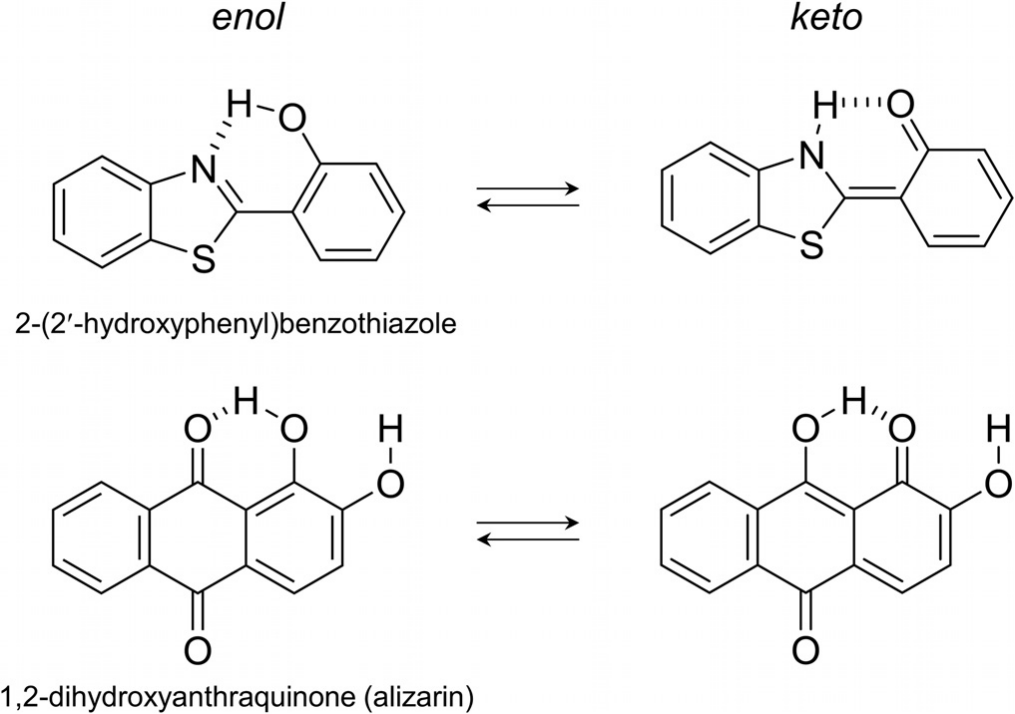
Examples of excited-state intramolecular proton transfer (ESIPT) reaction.

Proton transfer reaction has been of great interest since it is considered as one of the fundamental chemical reactions in many chemical and biological systems. Ultrafast proton transfer reactions either intra- or intermolecular occurring in the excited states have been extensively studied by many time-resolved electronic and vibrational spectroscopic techniques.^1–14^ For example, ultrafast proton transfer reactions of ∼30 fs time constant have been observed from 10-hydroxybenzo[*h*]quinolone (HBQ) and 2–(2-hydroxyphenyl)benzothiazole by femtosecond transient absorption spectroscopy.^6,13^ Recently, Joo and co-workers observed the rate constant of the excited-state intramolecular proton transfer (ESIPT) of HBQ and 1-hydroxy-2-acetonaphthone as fast as ∼12 fs by the fluorescence upconversion technique.^2,15,16^ It is considered that the tautomerization between the hydroxyl proton and the adjacent heterocyclic N atom or carbonyl group generally occurs on time scales of several tens of femtoseconds.^6,16,17^ The ESIPT reactions, including the tautomerization of 1,2-dihydroxyanthraquinone (alizarin), are displayed in Fig. 1. Large Stokes' shifts in the emission from the “proton-transferred (PT)” tautomer are frequently observed from the dyes with ESIPT. The acidity change of the proton donating group upon photoexcitation is generally considered as the driving force for the intra- or intermolecular proton transfers in the excited states.^18–20^ Intermolecular proton transfers have also been extensively studied by many time-resolved spectroscopic methods.^18,21^ The deprotonation of a photoacid, 8-hydroxypyrene-1,3,6-trisulfonate occurs with complicated excited-state dynamics including the formation and dissociation of the contact ion pair, which is also strongly coupled to solvent relaxations.^1,22^

Femtosecond stimulated Raman spectroscopy (FSRS) has been successfully applied to many photophysical and photochemical processes occurring on ultrafast time scales due to its high spectral and temporal resolutions.^23–26^ Among numerous recent works adopting FSRS, intramolecular charge transfer of 4-(dicyanomethylene)-2-methyl-6–(4-dimethylaminostyryl)-4H-pyran accompanying the rotation of the electron donor group has been clearly explained with the coexistence of the locally-excited and charge-transferred states in the first singlet excited state.^27^ Compared to previous time-resolved spectroscopic works,^28–30^ the recent FSRS results clearly resolved multiple vibrational bands of the molecules in the excited state with an ∼10 cm^−1^ spectral resolution sufficient for Raman spectroscopy. The detailed Raman spectrum of the chromophore in the excited states will be essential in the structural identification of reactants and products via theoretical simulation works by the time-dependent density functional theory method.

Solvation dynamics play crucial roles in various chemical reactions including charge transfer and proton transfer reactions and have been extensively studied by numerous experimental and theoretical methods.^31–34^ The solute-solvent interactions connect the reactant and product states by opening energetically favored reaction channels. Ultrafast responses of the polar solvent around the polar chromophore during a chemical reaction (called polarization relaxation) provide local and pertinent information to the chemical reaction. Among many experimental methods including the optical Kerr effect and photon-echo spectroscopy, the time-dependent fluorescence Stokes shift method is one of the most frequently used experiments.^5,32,35,36^ Recently, Vauthey and co-workers reported solvation dynamics of a number of small organic molecules in various solvents by fluorescence upconversion spectroscopy, where the solvation dynamics showed a strong dependence on the solute-solvent interactions such as hydrogen bonding in addition to the dynamics properties of the solvent itself.^35^

Time-resolved vibrational spectroscopy is another useful tool for the study of solvation dynamics. The vibrational modes of solute and solvent molecules, which are strongly coupled to the solute-solvent interactions, are perturbed by the excitation of the solute molecules. Nibbering and co-workers reported an ultrafast appearance of the free ν_C=O_ mode of Coumarin 102 and the free ν_OH_ mode of phenol upon photoexcitation, which implies instantaneous breakage of hydrogen bonding between the solute and solvent molecules.^37,38^ The solvation dynamics including the ultrafast hydrogen bond cleavage has also been observed in a recent time-resolved stimulated Raman work by Fang and co-workers.^39^

Alizarin is one of the model compounds for the ESIPT between the hydroxyl and adjacent carbonyl groups.^40–44^ The structural changes of alizarin from a 9,10-keto (locally-excited; LE) to a 1,10-keto (PT) form with a huge Stokes' shift of ∼7000 cm^−1^ have been investigated recently by FSRS, where the ultrafast ESIPT of a 70–80 fs time constant has been clearly evidenced in the population and relaxation dynamics of the major skeletal vibrations of ν_C=C_ and ν_C=O_.^45^ The excited-state dynamics of alizarin have also been investigated by transient absorption spectroscopy, where two distinct kinetic components (1.1 and 83.3 ps in DMSO, 3.4 and 84 ps in ethanol, and 1.2–2.5 and 58–59 ps in methanol) were observed in addition to the faster kinetic component (0.3–0.5 ps in ethanol and methanol).^45,46^ The slower 60–80 ps dynamics of alizarin was considered as the population decay of the PT conformer state, but the unambiguous assignments for the fast kinetic components as the ESIPT dynamics or the vibrational relaxation were not made by transient absorption measurements.

DMSO forms relatively weak self-interactions between neighboring molecules in the liquid phase, which is represented as the sub-bands for “free,” “aggregated,” and “hydrogen-bonded” DMSO in the vibrational spectrum of the ν_S=O_ and ν_CSC_.^47–50^ DMSO forms the intermolecular hydrogen bonding with water or glucose via the weak proton acceptor group of S =O.^50,51^ The hydrogen-bonding interactions result in the spectral changes of the vibration modes of DMSO. The ν_S=O_ shows strong red-shifts from 1058 (free) and 1044 cm^−1^ (aggregated) to 1022 (1-hydrogen-bonded) and 1012 cm^−1^ (2-hydrogen-bonded), and the ν_CSC_ shows blue-shifts from 663 (free) and 668 cm^−1^ (aggregated) to 676 cm^−1^ (hydrogen-bonded). The DMSO solution of highly concentrated lithium salts also showed similar spectral changes through cationic solvations.^52–55^

In this paper, the ultrafast ESIPT reaction of alizarin and the solvent dynamics of DMSO in the ν_S=O_ and ν_CSC_ modes have been investigated by FSRS. The ultrafast structural changes of alizarin induce instantaneous changes in the solvation shell of DMSO, which in return perturbs the intermolecular interactions between neighboring DMSO molecules. The spectral changes in the solvent vibrational modes of DMSO subsequent to the ESIPT of alizarin will be confirmed by several control experiments with similar hydroxyanthraquinone without the ESIPT and with deuterated alizarin at hydroxyl positions.

## EXPERIMENTAL

### General

Alizarin purchased from Sigma-Aldrich (St. Louis, MO, USA), and 1,2,4-trihydroxy-anthraquinone (purpurin) and curcumin purchased from TCI (Tokyo, Japan) were used without further purification. Alizarin and purpurin solutions of 20 mM concentration in DMSO (Daejung Chemicals and Metals, Siheung, Korea) were used for stimulated Raman measurements. The acetone solution of alizarin (40 mM) was mixed with 10 equivalent of deuterium oxide (Cambridge Isotope Laboratory, Tewksbury, MA, USA) and stirred for 48 h at room temperature. The solution was dried under reduced pressure, and the residue was dissolved in acetone. Deuteration with deuterium oxide was repeated, and the deuterated alizarin was obtained with >85% deuterium incorporation. However, alizarin-d_2_ was unstable in the solution phase due to the hygroscopic property of DMSO. We added an excess amount (0.33%, v/v) of deuterium oxide in the DMSO solution of alizarin-d_2_ in order to minimize the back reaction into alizarin-h_2_ during the transient Raman measurements. A 0.5 mm thick quartz flow cell and a peristaltic pump were used to recirculate the samples to minimize photodamage from the intense laser pulses.

### FSRS setup

A home-built time-resolved Raman setup based on a 1 kHz Ti:sapphire regenerative amplifier (50 fs, 805 nm) was used for the FSRS measurements.^45^ The broadband Raman probe generated from supercontinuum generation in a YAG window (4 mm thick; Newlight Photonics, Toronto, CA) and the narrowband Raman pump from a grating filter with a 1200 g/mm grating (∼750 nJ/pulse) were combined at the sample for the stimulated Raman processes. An actinic pump at 403 nm (∼350 nJ/pulse) was generated by second-harmonic generation in a BBO crystal (θ = 29.2°, 0.1 mm thick; A-Star Photonics, Fuzhou, China) and compressed by a pair of chirped mirrors (−25 ± 10 fs^2^ group delay dispersion; Layertec GmbH, Mellingen, Germany). A multichannel CCD detector at 1 kHz readout (PIXIS 100; Princeton Instruments, Trenton, NJ, USA) was used to measure very small (2 × 10^−5^ level) changes in the probe intensity. The temporal resolution of the FSRS setup was evaluated by the optical Kerr effect measurements, where the polarization rotation of the Raman probe pulses by the actinic pump pulses was recorded as a function of the time delay between two pulses.^44^ The cross correlation time between these two pulses was measured as 60 fs with a 150 *μ*m thick cover glass and 93 fs with a 0.5 mm quartz flow cell filled with solvent DMSO (see Fig. S1 in the supplementary material).

## RESULTS AND DISCUSSION

### Ultrafast intramolecular proton transfer of alizarin

**FIG. 2.**
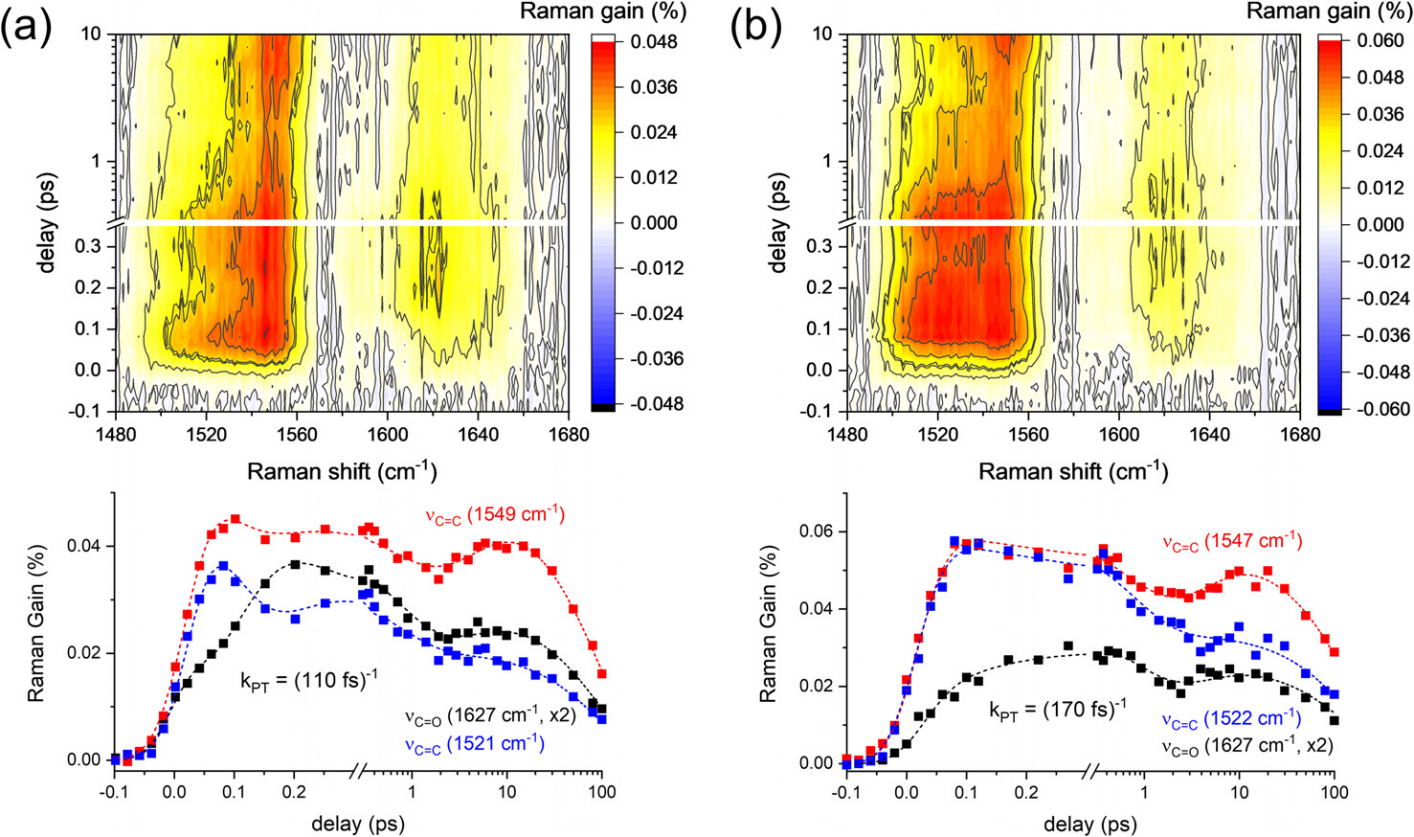
FSRS of (a) alizarin-h_2_ and (b) alizarin-d_2_ in DMSO solution with 403 nm excitation. The kinetic traces of the ν_C=C_ and ν_C=O_ modes were compared in the lower panels.

The FSRS of alizarin-h_2_ and alizarin-d_2_ in DMSO solution obtained with 403 nm excitation are compared in Fig. 2, where the transient skeletal vibrations of ν_C=C_ and ν_C=O_ in the singlet excited state represented the ultrafast reaction dynamics of ESIPT. In our previous report with alizarin-h_2_, the ESIPT of alizarin was shown to occur with a 70–80 fs time constant by the population growth, opposite peak shifts, and bandwidth changes of both skeletal vibrations.^45^ In this work, we further updated the pulse compression of the actinic pump and was able to monitor the differences in the excited-state kinetics of ν_C=C_ and ν_C=O_ bands. The surface plot and the corresponding kinetic traces for alizarin-h_2_ are shown in Fig. 2(a), and time-resolved spectra at selected time delays are also shown in Fig. S2(a) in the supplementary material. Transient Raman band intensities for the ν_C=C_ (at 1521 and 1549 cm^−1^) and ν_C=O_ (at 1627 cm^−1^) of alizarin-h_2_, and the ν_C=C_ (at 1522 and 1547 cm^−1^) and ν_C=O_ (at 1627 cm^−1^) of alizarin-d_2_ were analyzed with the parallel kinetic model with a number of convoluted functions between the instrument response function (IRF) Gaussian and exponential function,

$${\text{GE}}_{i}\left(t\right)=A_{i}\;\text{exp}\;\left(\frac{\omega^{2}}{2\tau_{i}^{2}}-\frac{t-t_{0}}{\tau_{i}}\right)\left[1-\mathrm{erf}\left(\frac{\omega^{2}-\tau_{i}(t-t_{0})}{\sqrt{2}\omega \tau_{i}}\right)\right],$$ (1)

where *A_i_* represents the amplitude, τ_*i*_ the exponential lifetime, and ω the width (variance) of a Gaussian function.^56^ We performed a global analysis for all the ν_C=C_ and ν_C=O_ modes of alizarin-h_2_ and alizarin-d_2_, where all the exponential lifetimes τ_*i*_ and the Gaussian width ω were shared over all the vibrational modes. The time zeros of each component *t*_0_ were determined separately from the temporal chirp measurements between the actinic pump and the Raman probe pulses.

However, we could not get satisfactory fit results with the 2–4 separate Gaussian-convoluted exponential functions GE_*i*_(*t*), especially for the case of alizarin-h_2_ due to the strong coherent oscillation patterns observed in all the vibrational modes. A damped oscillation component with a separate damping lifetime τ_D_ and the oscillation time constants τ_osc,ν_ was added in the global analysis,

$${\text{OSC}}_{\nu }\left(t\right)=A_{\mathrm{osc},\nu }\;\text{exp}\;\left(\frac{\omega^{2}}{2\tau_{D}^{2}}-\frac{t-t_{0}}{\tau_{D}}\right)\left[1-\mathrm{erf}\left(\frac{\omega^{2}-\tau_{D}(t-t_{0})}{\sqrt{2}\omega \tau_{D}}\right)\right]\cdot \;\text{sin}\;\left(2\pi \frac{t-t_{0}}{\tau_{\mathrm{osc},\nu }}\right).$$ (2)

After numerous trial and errors, we found out that the fit results obtained with a common time constant for τ_D_ and τ_1_ (110 fs) and the fixed phase values (0 or 180°) for the sine functions well matched the measured kinetics for the ν_C=C_ and ν_C=O_ modes of alizarin-h_2_. The oscillation time constants τ_osc,ν_ of the ν_C=C_ and the ν_C=O_ modes of alizarin-h_2_ were found as 0.21–0.27 ps, which slightly varies for the skeletal vibrational modes. The fast Fourier transformation analysis on the damped oscillation fit results of these vibrational modes revealed the vibrational frequencies of 121–156 cm^−1^ for the skeletal vibrational modes of alizarin-h_2_, which can be considered as the vibrational frequencies for the intramolecular couplings of alizarin-h_2_ between the carbonyl and hydroxyl groups or the intermolecular coupling via the hydrogen-bonding network of solvent DMSO molecules (see Fig. S3 in the supplementary material).^26,57–60^ The fit results of alizarin-h_2_ shown in Fig. 2(a) are summarized in Table I.

**TABLE I.** The excited-state kinetics for ν_C=C_ and ν_C=O_ modes of alizarin-h_2_.

	Shared parameters				
	*ω*	*τ_1_* (=*τ_D_*)	*τ_2_*	*τ_3_*	*τ_4_*
Fit results (ps)	0.04 ± 0.00	0.11 ± 0.02	0.74 ± 0.10	5.08 ± 1.13	94.4 ± 4.3

Vibrational mode	Individual parameters^a^						
	*t*_0_ (fs)	*A_1_*	*A_2_*	*A_3_*	*A_4_*	*A_osc,ν_*	*τ_osc,ν_* (ps)
ν_C=C_ (1521 cm^−1^)	10 ± 1	-	6 ± 0	-	10 ± 0	6 ± 1	0.24 ± 0.01
ν_C=C_ (1549 cm^−1^)	12 ± 1	-	8 ± 1	−9 ± 1	24 ± 1	2 ± 1	0.21 ± 0.03
ν_C=O_ (1627 cm^−1^)	18 ± 1	−8 ± 1	11 ± 1	−4 ± 1	14 ± 0	-7 ± 1	0.27 ± 0.02

^a^ All the amplitudes, *A_i_* and *A_osc,ν_*, are displayed in thousandths.

The ν_C=O_ band of alizarin-h_2_ at 1627 cm^−1^ showed a rise with *τ*_1_ = 110 fs, while the intensities of two ν_C=C_ modes at 1521 and 1549 cm^−1^ showed no major decay. However, strong coherent oscillation patterns with the same time constant, *τ*_D_ = 110 fs, were observed in the intensities of all three skeletal vibration modes of alizarin-h_2_. Furthermore, the coherent oscillation signals in the ν_C=C_ modes appeared to be out of phase with those in the ν_C=O_ mode, as shown in Fig. S3 in the supplementary material. Thus, the 110 fs time constant is considered as the ESIPT process of alizarin-h_2_ in the excited state since this time constant is commonly observed in the population growth of the ν_C=O_ mode and the coherent oscillations of the skeletal vibrational modes of ν_C=C_ and ν_C=O_. The 110 fs ESIPT time constant is slightly larger than the value (70–80 fs) estimated from our previous study.^45^ It seems that the coherent oscillation signals contained in the skeletal vibrational modes of alizarin-h_2_ may have resulted in a little smaller time constant for the ESIPT.

The ultrafast structural changes accompanying the coherent nuclear wave-packet motions perpendicular to the reaction coordinate have been numerously observed in many chromophores, especially in the strongly coupled vibrational modes to the reaction coordinate normal mode.^61–64^ We showed that the ν_C=O_ mode of alizarin-h_2_ at 1627 cm^−1^ represents the excited-state vibration of the PT conformer and that two ν_C=C_ modes of alizarin-h_2_ at 1521 and 1549 cm^−1^ are strongly coupled to the ESIPT reaction coordinate. The coherent nuclear wave-packet motions appearing in the skeletal vibrational modes of ν_C=C_ and ν_C=O_ modes with the same damping lifetime as the ESIPT dynamics of 110 fs and also with the same or opposite phase between the ν_C=C_ and ν_C=O_ modes may reveal that the ESIPT of alizarin occurs via a transition state of hypothetical six-membered ring including the carbonyl and the adjacent hydroxyl groups. The existence of the transition state for the ESIPT process was first proposed in our previous report where the ν_C=C_ and ν_C=O_ modes of alizarin-h_2_ with the opposite peak shifts during the ESIPT were interpreted as the structural changes of the resonant transition state.^45^ In this report, we added another piece of experimental evidence for the vibrational coupling between the ν_C=C_ and ν_C=O_ modes of alizarin-h_2_, which may be considered inevitable in the ESIPT process.

All the skeletal vibrational modes of alizarin-h_2_ showed the vibrational relaxation components of 0.7 and 5.1 ps in the potential surface of the PT conformer and the population dynamics of 94 ps, which is similar to the 1.1 and 83.3 ps dynamics from transient absorption measurements.^45^ These relaxation dynamics of alizarin-h_2_ after the ESIPT will be compared to the solvation dynamics of DMSO in the subsection of “Solvation dynamics of DMSO.”

The surface plot and the corresponding kinetic traces of the ν_C=C_ and ν_C=O_ bands in the FSRS of alizarin-d_2_ obtained with the 403 nm excitation are shown in Fig. 2(b). Time-resolved Raman spectra of alizarin-d_2_ at selected time delays are also presented in Fig. S2(b) in the supplementary material. Transient Raman band intensities of ν_C=C_ and ν_C=O_ of alizarin-d_2_ were successfully fit without any significant damped oscillation component. The time constant for the ESIPT process of alizarin-d_2_ was obtained as τ_1_ = 170 fs, which is clearly represented in the rise of the ν_C=O_ band at 1547 cm^−1^. The ESIPT process becomes slower (τ_1_ = 110 → 170 fs) upon the deuteration of hydroxyl proton. The similar kinetic isotope effects were reported from HBQ and quercetin, where the ESIPT rate constant increased 1.6–2 times upon deuteration.^10,15^ Two ν_C=C_ modes at 1522 and 1547 cm^−1^ do not show any decay of intensities during the ESIPT process of τ_1_ = 170 fs, while presented the vibrational relaxation components of 0.8 and 4.4 ps in the potential surface for the PT conformer state. These relaxation dynamics of alizarin-d_2_ after the ESIPT will also be compared to the solvation dynamics of DMSO in the later section. The population dynamics of 150 ps was observed from all the skeletal vibrational modes of alizarin-d_2_. The fit results for alizarin-d_2_ shown in Fig. 2(b) are summarized in Table II.

**TABLE II.** The excited-state kinetics for ν_C=C_ and ν_C=O_ modes of alizarin-d_2_.

	Shared parameters				
	*ω*	*τ_1_*	*τ_2_*	*τ_3_*	*τ_4_*
Fit results (ps)	0.04 ± 0.00	0.17 ± 0.07	0.80 ± 0.11	4.44 ± 1.49	152 ± 12
	Individual parameters^a^				
vibrational mode	*t*_0_ (fs)	*A_1_*	*A_2_*	*A_3_*	*A_4_*
ν_C=C_ (1522 cm^−1^)	10 ± 1	-	13 ± 1	–	17 ± 0
ν_C=C_ (1547 cm^−1^)	12 ± 1	-	13 ± 2	−10 ± 2	27 ± 1
ν_C=O_ (1627 cm^−1^)	18 ± 1	−11 ± 2	11 ± 3	−4 ± 2	13 ± 1

^a^ All the amplitudes, *A_i_*, are displayed in thousandths.

### Transient stimulated Raman spectra of DMSO

**FIG. 3.**
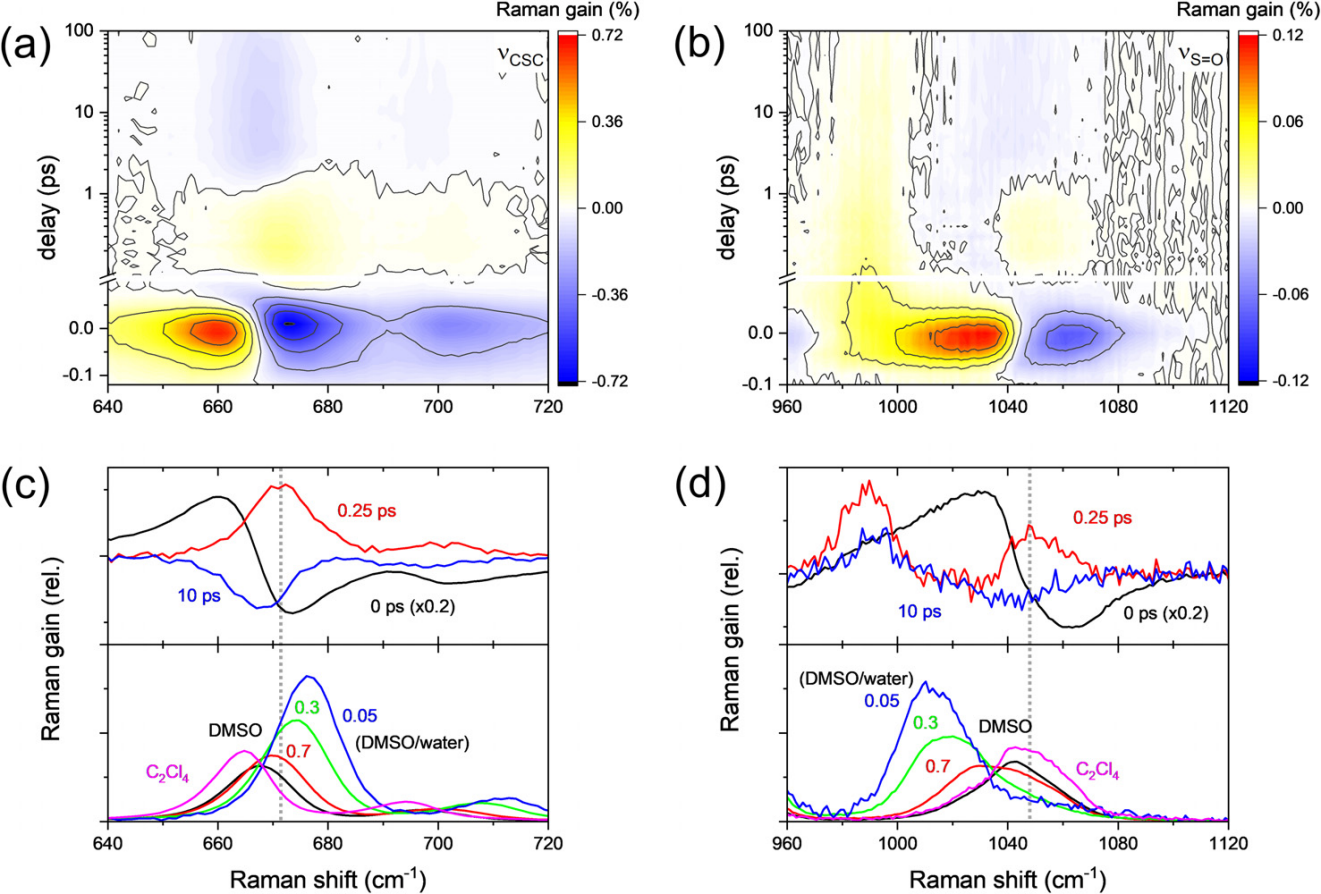
The surface plots for (a) ν_CSC_ and (b) ν_S=O_ of DMSO in the FSRS of alizarin-h_2_ with 403 nm excitation; the transient stimulated Raman spectra of (c) ν_CSC_ and (d) ν_S=O_ modes at 0.0, 0.25, and 10 ps (top) were compared with the Stokes Raman spectra of DMSO obtained from the C_2_Cl_4_ (1:1) solution, DMSO: water mixtures of (0.05:1, 0.3:1, and 0.7:1), and pure DMSO (bottom).

Figures 3(a) and 3(b) show the surface plots of the ν_CSC_ and ν_S=O_ modes of DMSO obtained with the FSRS of alizarin-h_2_ upon 403 nm excitation. In Figs. 3(c) and 3(d), the transient stimulated Raman spectra at 0.0, 0.25, and 10 ps from the surface plots in panels (a) and (b), respectively, were compared with the Stokes Raman spectra of DMSO dissolved in nonpolar C_2_Cl_4_ solvent, pure DMSO, and binary mixtures of DMSO: Water (*f*_DMSO_ = 0.7, 0.3, and 0.05).

The dispersive spectral patterns around the peak center of each vibrational mode at zero delay time shown in Figs. 3(c) and 3(d) at a 0 ps delay were observed in all the solvent vibrational modes of ν_CSC_ (symmetric at 667 and asymmetric at 698 cm^−1^), ν_S=O_ (1042 cm^−1^), and δ_CH3_ (at 953 and 1417 cm^−1^; not shown). These dispersive signals often called cross-phase modulation (CPM) artifacts originate from the nonlinear optical processes between the actinic pump and the Raman probe pulses occurring inside the solvent medium.^65^ The CPM signals in the FSRS of alizarin-h_2_ (or -d_2_) showed Gaussian profiles of a 95 fs pulse width [see Fig. S8(a) in the supplementary material], which is quite similar to the instrument response function of FSRS measurements.

Other than the CPM artifacts, two separate transient features in the solvent vibrational modes were observed in the FSRS results with alizarin. One is the long-lasting negative features centered at 668 and 1042 cm^−1^ for the ν_CSC_ and ν_S=O_ modes, respectively [see transient Raman spectra at a 10 ps delay shown in Figs. 3(c) and 3(d)]. These negative signals are considered as the vibrationally “hot” Stokes Raman signal of DMSO resulting from the local heating of the actinic pump pulses.^66^ The negative Stokes Raman signals of DMSO become much larger with increased actinic pump energy (see Fig. S4 in the supplementary material) and disappear with much longer time constants of 100–300 ps, which is compatible to the vibrational relaxation time of liquids.^67,68^

Between the CPM artifacts and the long-lasting negative Stokes Raman signals of DMSO, we observed ultrafast positive bands for both the ν_CSC_ and ν_S=O_ (centered at 672 and 1050 cm^−1^, respectively) within time delays of 0.1 and 2 ps. Transient stimulated Raman spectra of DMSO at a 0.25 ps delay shown in Figs. 3(c) and 3(d) represent the ultrafast Raman bands of DMSO. These bands are clearly different from the ground state spectra of the ν_CSC_ and ν_S=O_ of pure DMSO [black lines in the lower panels of Figs. 3(c) and 3(d)] in terms of the peak positions or band shapes. The ν_CSC_ and ν_S=O_ bands of pure DMSO at 668 and 1042 cm^−1^ represent mostly the “aggregated” species of DMSO. The transient difference spectrum of the ν_CSC_ at 672 cm^−1^ is rather similar to the spectrum of a 1:1 DMSO/water mixture or highly concentrated solutions of lithium perchlorate or tetrafluoroborate.^52,53,55^ The transient band of the ν_S=O_ at 1050 cm^−1^ is similar to the “free” DMSO spectrum obtained from a 1:1 DMSO/C_2_Cl_4_ mixture, but clearly different from the solvated spectrum of ν_S=O_ obtained with lithium cations.^53,54^

We doubt that these bands originate from any temporal effect due to actinic or Raman pump pulses based on the following reasons. These bands appear in <100 fs after the pulse and disappear with 3–5 ps time scales, which is quite similar to the vibrational relaxation dynamics (0.7 and 5.1 ps for alizarin-h_2_ and 0.8 and 4.4 ps for alizarin-d_2_) of the product 1,10-keto tautomer of alizarin in the excited state. The decay of these transient signals might be too fast to be considered as any thermal effect of the solvent medium. Second, a similar transient band was not observed for the δ_CH3_ mode of DMSO (see Fig. S5 in the supplementary material), which clearly excludes any possibility of thermal issues. Therefore, we propose that these transient Raman signals of DMSO in the ν_CSC_ and ν_S=O_ modes result from the structural changes of DMSO molecules in and near the solvation shells. Other bands at 987 cm^−1^ of alizarin-h_2_ [Figs. 3(b) and 5(c)] and 962 cm^−1^ of alizarin-d_2_ [Fig. 5(d)] near the ν_S=O_ mode of DMSO are considered as the ν_CC_/δ_CH_ modes.^69,70^ These bands showed quite similar kinetics to those of the ν_C=C_ modes at 1521 and 1549 cm^−1^ for alizarin-h_2_ [Fig. 2(a)] and at 1522 and 1547 cm^−1^ for alizarin-d_2_ [Fig. 2(b)].

**FIG. 4.**
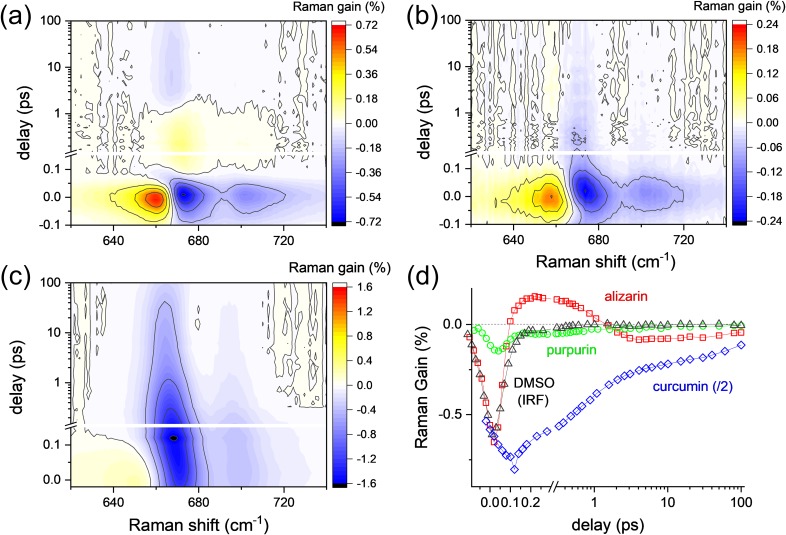
The ν_CSC_ band of DMSO in the FSRS of (a) alizarin-h_2_, (b) purpurin, and (c) curcumin with 403 nm excitation; (d) solvation dynamics for the ν_CSC_ of DMSO in alizarin, purpurin, and curcumin solutions were compared with that of CPM artifacts (IRF).

To further explore the origin of these transient Raman signals of DMSO, we measure the FSRS of two dyes purpurin and curcumin in DMSO solution with 403 nm excitation. It is known that purpurin does not exhibit proton transfer in the excited state similarly as 1,4-dihydroxyanthraquinone (quinizarin).^9,71,72^ Figure 4(b) clearly shows the disappearance of the ultrafast transient Raman signal of ν_CSC_ at 672 cm^−1^ and ν_S=O_ at 1050 cm^−1^ with purpurin, where the ultrafast structural changes of purpurin upon intramolecular proton transfer are not expected. The transient Raman spectra of ν_S=O_ with purpurin and curcumin are shown in Fig. S6 in the supplementary material.

In addition, transient Raman spectra for the ν_CSC_ and ν_S=O_ of DMSO were measured with curcumin, where much stronger solvation with hydrogen bonding is expected.^73^ As shown in Fig. 4(c), transient Raman spectra for the ν_CSC_ of DMSO in curcumin solution showed much stronger negative bands in 670–680 cm^−1^, which is even larger than the CPM artifact signals. The ν_CSC_ band showed two solvation dynamic components of 90 fs and 2.3 ps from the solvent cross correlation function analysis, which is compatible with the previously reported dynamics by transient absorption and fluorescence upconversion measurements and molecular dynamics simulations.^59,73,74^ It is apparent that the solvation dynamics of DMSO in alizarin and purpurin are quite different from the case of curcumin, where the strong couplings via hydrogen bonding affect the ground state vibrational modes of solute (curcumin) and solvent (DMSO) molecules with the similar solvation dynamics (not shown in this work and will be prepared for the subsequent publication). Thus, transient Raman bands of DMSO (ν_CSC_ at 672 cm^−1^ and ν_S=O_ at 1050 cm^−1^) observed with alizarin are understood as originating from the instantaneous changes in the solvation of DMSO around alizarin with hydrogen-bonding-like interactions. The population dynamics for the transient Raman bands of ν_CSC_ in alizarin, purpurin, and curcumin solutions were compared with that of CPM artifacts in Fig. 4(d).

### Solvation dynamics of DMSO

**FIG. 5.**
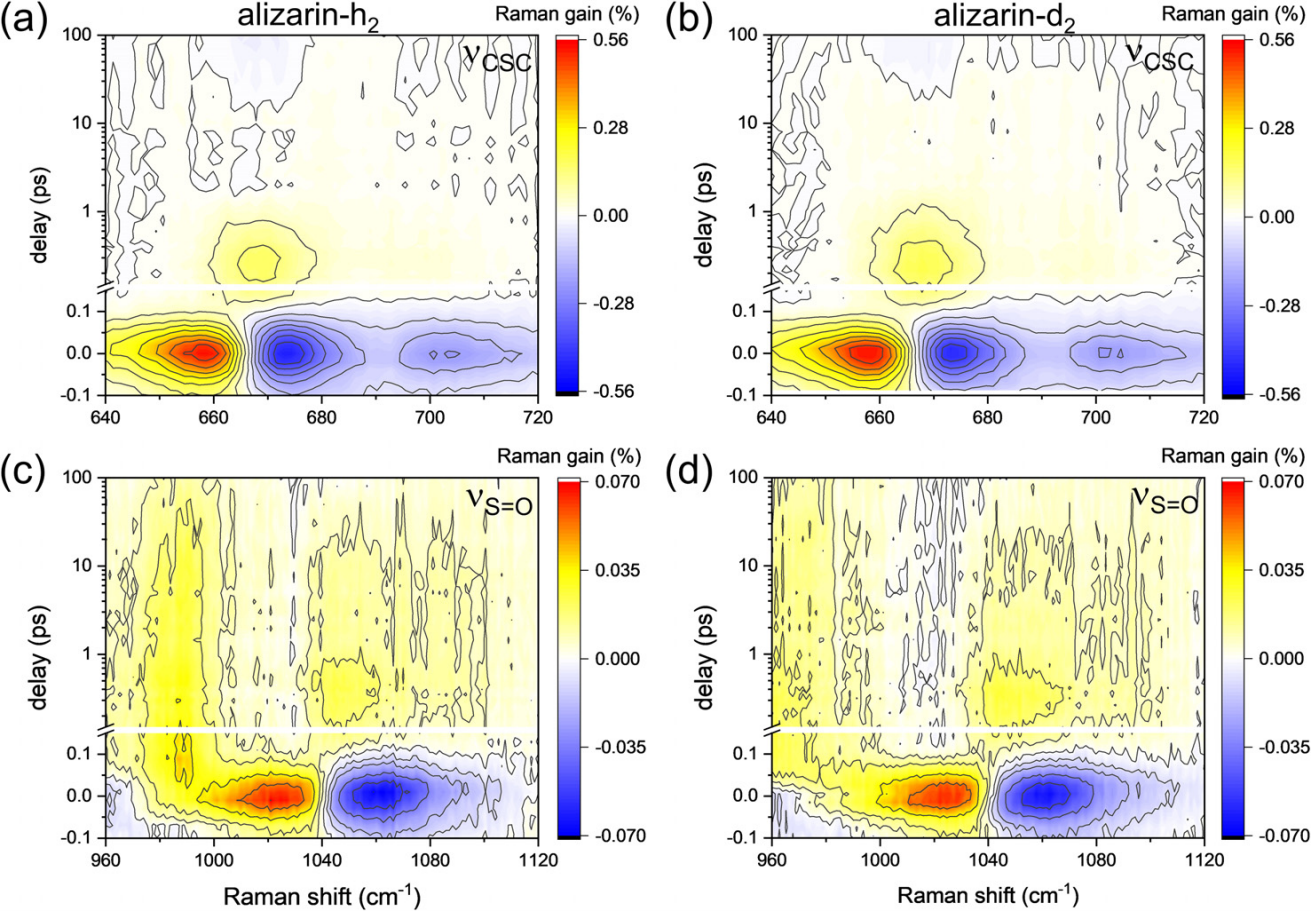
The solvent vibrational modes of DMSO in the FSRS with 403 nm excitation; (a) ν_CSC_ and (c) ν_S=O_ bands obtained with alizarin-h_2_, (b) ν_CSC_ and (d) ν_S=O_ bands obtained with alizarin-d_2_.

To further explore the solvation dynamics of DMSO upon the ESIPT of alizarin, we compared the transient spectral changes of ν_CSC_ and ν_S=O_ modes of DMSO upon the deuteration of hydroxyl protons of alizarin-h_2_ in Fig. 5. To minimize the negative vibrationally hot Stokes Raman signals of DMSO, the pulse energies for both the actinic and Raman pumps were decreased for the measurement of solvent vibrational spectra upon the ESIPT. The transient solvent bands of DMSO appearing in a 0.1–1.0 ps delay range and at 672 (ν_CSC_) and 1050 cm^−1^ (ν_S=O_) showed no major kinetic difference between alizarin-h_2_ and alizarin-d_2_. Unfortunately, the existence of the nonlinear CPM bands around zero time delay makes it difficult to compare the formation dynamics of the ultrafast transient solvent bands (ν_CSC_ and ν_S=O_) of DMSO. The nonlinear CPM signals obtained together with the FSRS of solute and solvent molecules were simulated from its own spectral and temporal profile and removed from the experimental data (refer to the supplementary material including Figs. S8 and S9).

**FIG. 6.**
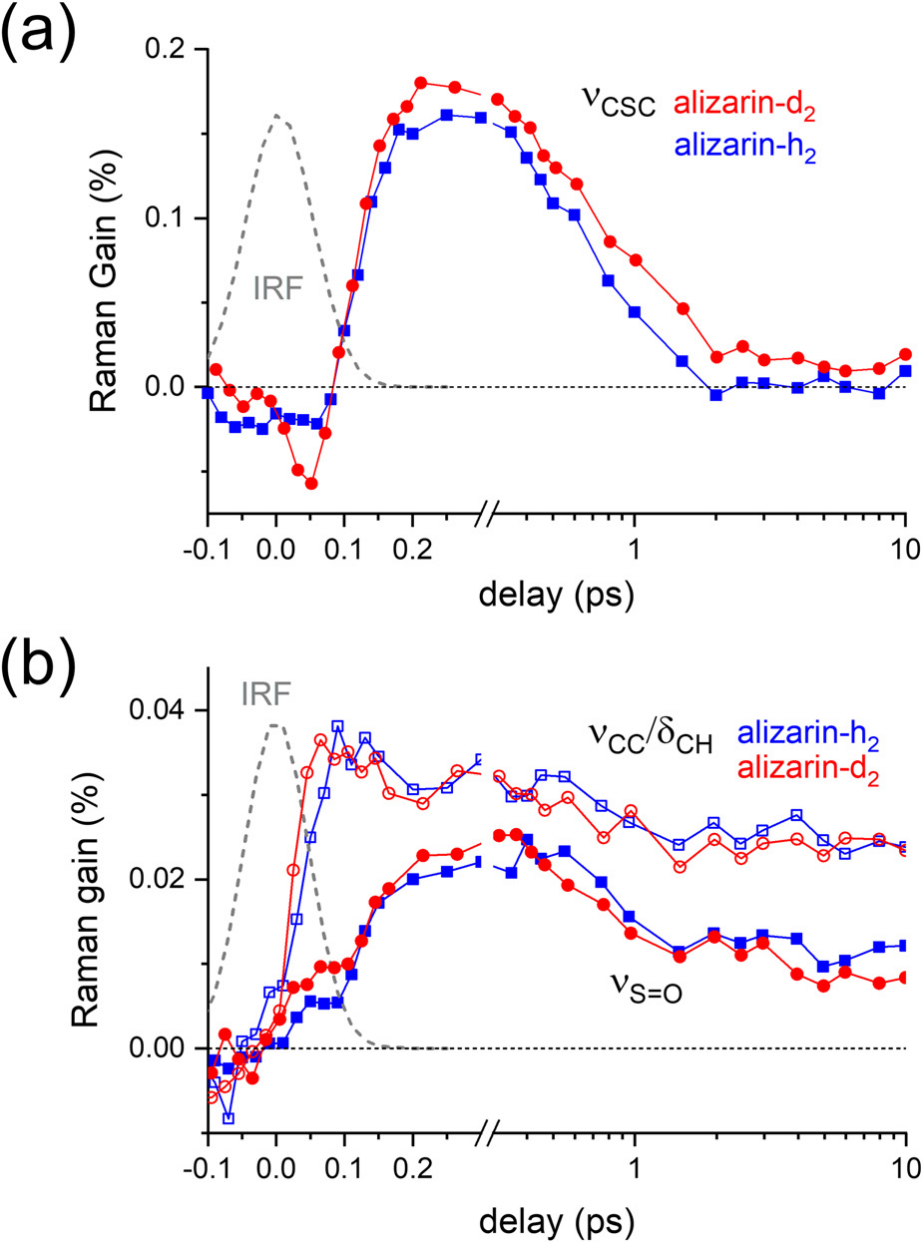
The kinetics of (a) ν_CSC_ and (b) ν_S=O_ of DMSO in the FSRS of alizarin-h_2_ and alizarin-d_2_ with 403 nm excitation. The kinetics of the ν_CC_/δ_CH_ band (at 987 and 962 cm^−1^) of alizarin-h_2_ and alizarin-d_2_, respectively, were compared as open symbols in panel (b) and the instrument response function (IRF) of FSRS measurements as dotted lines.

The kinetic trances for the ν_CSC_ (672 cm^−1^) and ν_S=O_ (1050 cm^−1^) of DMSO without the CPM signals obtained with the alizarin-h_2_ and alizarin-d_2_ were compared in Fig. 6. The kinetic trances of the ν_CC_/δ_CH_ (987 and 962 cm^−1^) near the ν_S=O_ band of alizarin-h_2_ and alizarin-d_2_, respectively, were also compared in Fig. 6(b). The kinetic traces for the ν_CSC_ and ν_S=O_ modes of DMSO showed no difference between the solutes of alizarin-h_2_ and alizarin-d_2_, while the dynamics for the appearance and disappearance of the ultrafast transient solvent bands showed a clear difference between the ν_CSC_ and ν_S=O_. The dynamics of the ν_CSC_ mode of DMSO showed a 60–70 fs rise strongly coupled to the excitation pulse and a 0.5–0.6 ps decay. On the other hand, the dynamics of ν_S=O_ showed a slower and secondary rise of 100–120 fs and multiexponential decays with a faster 0.4–0.5 ps and a slower ∼50 ps time constant. The ν_CSC_ mode of DMSO showed an almost instantaneous response with the excitation of solute molecules, and no kinetic difference between the results with alizarin-h_2_ and alizarin-d_2_ (compared to the 110 and 170 fs dynamics for the ESIPT of alizarin-h_2_ and alizarin-d_2_, respectively) was observed in our measurements. On the other hand, the ν_S=O_ mode showed slower increases of 100–120 fs upon the excitation of solute molecules, which is quite similar to the ESIPT dynamics of alizarin-h_2_. No significant kinetic differences in the rise of the ν_S=O_ mode with alizarin-h_2_ and alizarin-d_2_ were either observed. The incomplete removal of the CPM artifact around time zero makes it difficult for us to differentiate the ultrafast responses in the solvent vibrational modes of DMSO with the ESIPT process of alizarin-h_2_ and alizarin-d_2_. It is interesting to note that the 0.5–0.6 ps decay of the transient ν_CSC_ band and 0.4–0.5 ps decay of the transient ν_S=O_ band are quite similar to the fast components of the relaxation dynamics for the solute vibrational modes of alizarin in the PT conformer state (0.7 ps for alizarin-h_2_ and 0.8 ps for alizarin-d_2_). Thus, we further confirm that the fast relaxation dynamics of alizarin solute vibrational modes are strongly related to the decay of the transient Raman bands for the ν_CSC_ and ν_S=O_ modes of DMSO. The slower relaxation dynamics of solute vibrational modes (5.1 ps for alizarin-h_2_ and 4.4 ps for alizarin-d_2_) were then assigned as the relaxation dynamics of solute vibrational modes along the potential surface of the PT conformer state.

Baiz and co-workers investigated the hydrogen-bonding dynamics in the mixtures of DMSO and water by FT-IR measurements and molecular dynamics simulations.^50^ The ν_S=O_ mode of DMSO strongly involved in the solvation and dimer formation was proposed to consist of several subbands including the “free,” “aggregated,” and “hydrogen-bonded” species. In our time-resolved Raman measurements, we observed the ultrafast solvent responses in the ν_S=O_ and ν_CSC_ modes of DMSO upon the ESIPT process of alizarin, especially in the species of free or aggregated. Furthermore, we presented the distinct dynamics for these two vibrational modes of DMSO both sensitive to the solvation and hydrogen bonding. The ν_S=O_ mode showed the ultrafast responses in the free or aggregated species with the relevant kinetics of the ESIPT process of the solute alizarin-h_2_ and alizarin-d_2_, while the ν_CSC_ modes of DMSO showed almost instantaneous responses, which can be understood merely as the breakage of the solvation (or hydrogen bonding) upon the laser excitation. However, no bleaching signals due to the solvation change were observed in the ν_CSC_ and ν_S=O_ modes of DMSO. Considering the low concentration of alizarin-h_2_ and alizarin-d_2_ (20 mM) compared to the bulk concentration of DMSO (14 M), a small portion of DMSO molecules would only be responsible for the solvation of alizarin molecules. Then, the hydrogen-bonded species of DMSO in the ground state Raman spectra of the ν_CSC_ and ν_S=O_ and the bleaching of the hydrogen-bonded mode in the FSRS results of alizarin-h_2_ and alizarin-d_2_ cannot be measured. Time-resolved Raman spectra of DMSO observed in both Stokes and anti-Stokes regions would provide more detailed information for the solvation dynamics of DMSO molecules upon the ESIPT of alizarin-h_2_ and alizarin-d_2_.

The disappearance of both the “transient” vibrational bands of DMSO was also slightly different. Both the ν_S=O_ and ν_CSC_ modes of DMSO showed an ∼0.5 ps decay, which can be interpreted as the relaxation of the transient molecular network of DMSO molecules and also as the vibrational relaxation of the ESIPT product of alizarin-h_2_ and alizarin-d_2_. In addition, the ν_S=O_ mode also showed a smaller and slower decay component of ∼50 ps, which shows that the ν_S=O_ mode of DMSO is more sensitive to the local changes in the molecular networks of DMSO or intermolecular interactions between DMSO molecules, in other words. Finally, we propose that the ν_S=O_ and ν_CSC_ modes of DMSO may be used as a vibrational sensor for the ultrafast chemical reactions of solute molecules. Further exploration of the ultrafast photoinduced processes in DMSO solution will substantiate our proposal.

## CONCLUSIONS

We report that the ESIPT of alizarin in DMSO solution can be observed both by the solute vibrational modes of alizarin and by the solvent vibrational modes ν_S=O_ and ν_CSC_ of DMSO. The ESIPT processes of alizarin-h_2_ and alizarin-d_2_ occurring on the ultrafast time scales of 110 and 170 fs, respectively, were successfully monitored by the ultrafast response in these solvent vibrational modes, especially for the “free” and “aggregated” species of DMSO. From the detailed kinetic analysis of the transient Raman results of DMSO upon the ESIPT process, the ν_S=O_ mode of DMSO was proposed to be more sensitive to the dynamics of ESIPT process and the local changes in the solvation or the intermolecular interactions between DMSO molecules. The observation of the excited-state reaction dynamics of solutes (often in lower concentrations) by the ultrafast spectral changes in the solvent vibrational modes of DMSO (with much larger spectral intensity) may extend the study of the excited-state processes to the systems where the solute vibrational modes cannot be measured due to low solute concentrations or inherently infinitesimal Raman cross sections.

## SUPPLEMENTARY MATERIAL

See the supplementary material for the instrument response function measurements of FSRS, transient Raman spectra of alizarin and coherent oscillation signals in ν_C=C_ and ν_C=O_ modes, vibrationally hot Stokes Raman spectra of DMSO, NMR and Raman spectra for the deuteration of alizarin, and the removal of nonlinear dispersive CPM artifacts from transient Raman data.
